# Prediction of osteoporosis from simple hip radiography using deep learning algorithm

**DOI:** 10.1038/s41598-021-99549-6

**Published:** 2021-10-07

**Authors:** Ryoungwoo Jang, Jae Ho Choi, Namkug Kim, Jae Suk Chang, Pil Whan Yoon, Chul-Ho Kim

**Affiliations:** 1 Department of Biomedical Engineering, University of Ulsan College of Medicine, Seoul, Republic of Korea; 2 University of Ulsan College of Medicine, Seoul, Republic of Korea; 3 Department of Convergence Medicine, University of Ulsan College of Medicine, Asan Medical Center, Seoul, Republic of Korea; 4 Department of Orthopaedic Surgery, Good Gangan Hospital, Busan, Republic of Korea; 5 Department of Orthopaedic Surgery, Asan Medical Center, University of Ulsan College of Medicine, Seoul, Republic of Korea; 6 Department of Orthopaedic Surgery, Chung-Ang University Hospital, Chung-Ang University College of Medicine, Seoul, Republic of Korea

**Keywords:** Medical research, Health care, Medical imaging

## Abstract

Despite being the gold standard for diagnosis of osteoporosis, dual-energy X-ray absorptiometry (DXA) could not be widely used as a screening tool for osteoporosis. This study aimed to predict osteoporosis via simple hip radiography using deep learning algorithm. A total of 1001 datasets of proximal femur DXA with matched same-side cropped simple hip bone radiographic images of female patients aged ≥ 55 years were collected. Of these, 504 patients had osteoporosis (T-score ≤ − 2.5), and 497 patients did not have osteoporosis. The 1001 images were randomly divided into three sets: 800 images for the training, 100 images for the validation, and 101 images for the test. Based on VGG16 equipped with nonlocal neural network, we developed a deep neural network (DNN) model. We calculated the confusion matrix and evaluated the accuracy, sensitivity, specificity, positive predictive value (PPV), and negative predictive value (NPV). We drew the receiver operating characteristic (ROC) curve. A gradient-based class activation map (Grad-CAM) overlapping the original image was also used to visualize the model performance. Additionally, we performed external validation using 117 datasets. Our final DNN model showed an overall accuracy of 81.2%, sensitivity of 91.1%, and specificity of 68.9%. The PPV was 78.5%, and the NPV was 86.1%. The area under the ROC curve value was 0.867, indicating a reasonable performance for screening osteoporosis by simple hip radiography. The external validation set confirmed a model performance with an overall accuracy of 71.8% and an AUC value of 0.700. All Grad-CAM results from both internal and external validation sets appropriately matched the proximal femur cortex and trabecular patterns of the radiographs. The DNN model could be considered as one of the useful screening tools for easy prediction of osteoporosis in the real-world clinical setting.

## Introduction

Osteoporosis is a common condition, especially in postmenopausal women; however, it often remains undetected until after fracture occurs. Early detection of osteoporosis is greatly important in preventing osteoporotic fractures. In the United States, the incidence of osteoporosis-related fractures is more than four times higher compared to that of stroke, heart attack, and breast cancer^1^, and based on the meeting report of the World Health Organization (WHO), osteoporotic fractures account for more hospital bed-days than those diseases in several high-income countries^2^. Hip fractures, one of the major osteoporotic fractures, are associated with limitations in ambulation, chronic pain and disability, loss of independence, and decreased quality of life, and 21%–30% of patients who have hip fracture die within 1 year^3^.

To date, the gold standard for osteoporosis diagnosis is the estimation of bone mineral density (BMD) in the hip and lumbar spine using dual-energy X-ray absorptiometry (DXA)^4^. According to the WHO guidelines, BMD ≤ 2.5 standard deviations below the young adult mean (T-score ≤  − 2.5) indicates osteoporosis, while a T-score at any site between − 1.0 and − 2.5 indicates low bone mass or osteopenia. Moreover, the US Preventive Services Task Force has recommended screening for osteoporosis with BMD testing to prevent osteoporotic fractures in women aged ≥ 65 years^3^.

However, even though DXA is the gold standard of osteoporosis diagnosis, it could not be widely used as a screening tool for osteoporosis because of its high cost and limited availability in developing countries^5,6^. To overcome these limitations, until now, great efforts have been made to develop a screening tool for osteoporosis. Quantitative ultrasonography is one of them, which has been developed as an alternative to DXA for screening osteoporosis. It is portable and more economical than DXA; however, it is insufficient to replace DXA as a screening tool for osteoporosis^6^. Furthermore, there are also various clinical risk assessment tools that have been developed to predict osteoporosis, including Fracture Risk Assessment Tool (FRAX), QFracture algorithm, Garvan Fracture Risk Calculator, and the Osteoporosis Self-assessment Tool^6,7^. These various risk assessment tools are easily accessible and useful; particularly, the FRAX calculator is a major achievement in terms of understanding and measuring fracture risk. However, a few limitations also exist, such as the lack of consideration of racial and ethnic group difference, especially those regarding body mass index and mortality rate^8^. Therefore, an advanced screening tool for osteoporosis is still needed in clinical practice.

Recently, artificial intelligence (AI) has been used for various medical imaging interpretation fields. Moreover, several studies attempted to apply AI technology for the development of a screening tool for osteoporosis. Based on simple radiographic data, there were a few trials to predict osteoporosis using machine learning or deep learning algorithm^4,9^. However, to the best of our knowledge, there were some limitations in the development of a real-world screening tool, such as inappropriate object detection results and extremely small sample size with methodological flaws.

This study aimed to predict osteoporosis from simple hip radiography by developing a deep neural network (DNN) model, which could be considered as a screening tool for osteoporosis by applying the latest techniques in the medical AI field, minimizing the previous limitations.

## Results

The mean age of all 1001 female patients was 72.5 ± 12.5 years (range 55.2–100.5). Moreover, 440 right hips and 561 left hips were included. The mean BMD of the total hip was 0.715 ± 0.162 g/cm^2^ (range 0.20–1.34), and the mean T-score of the total hip was − 2.2 ± 1.3 (range − 6.5–3.1).

### Validation of the deep learning model performance

The confusion matrix of the trained neural network applying the 101 test sets is shown in Table 1. Our final DNN model showed an overall accuracy of 81.2%, sensitivity (recall) of 91.1%, and specificity of 68.9%. The positive predictive value (PPV), which indicates “precision,” was 78.5%, and the negative predictive value (NPV) was 86.1%.

**Table 1:** Confusion matrix of the final model.

	Predicted OP	Predicted nonOP	Total
Real OP	51	14	65
Real nonOP	5	31	36
Total	56	45	101

*OP* osteoporosis.

**Figure 1:**
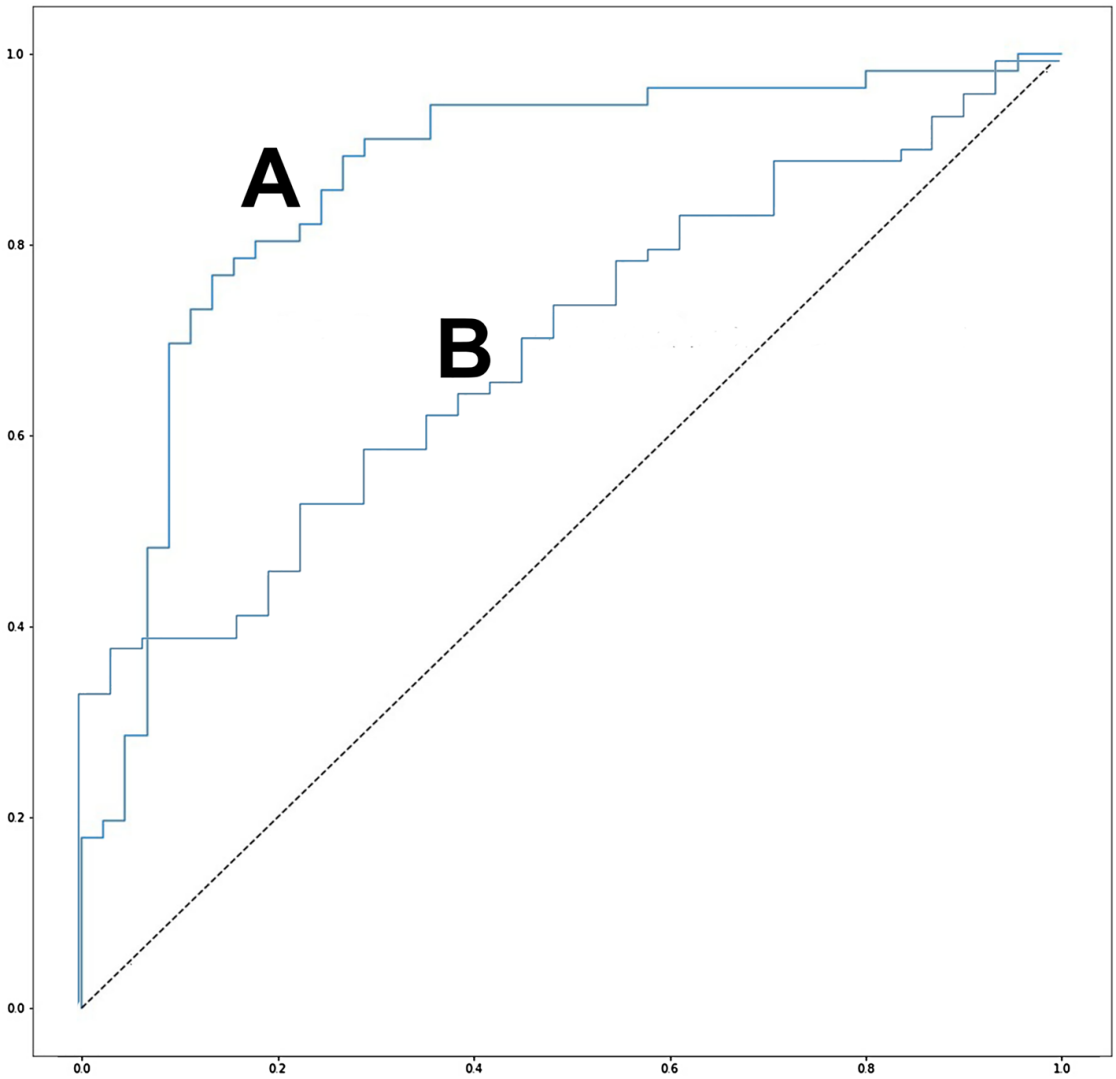
ROC curves of our osteoporosis prediction algorithm. The ROC curve of internal validation with 101 validation datasets is shown in (**A**) with AUC value of 0.867, and the ROC curve of external validation with 117 datasets is shown in (**B**) with AUC value of 0.700.

Furthermore, to evaluate the performance of our classification model, we drew the receiver operating characteristic (ROC) curve shown in Fig. 1. The area under curve (AUC) value was 0.867, which indicates excellent performance^10^.

### Visualization of model performance: gradient-based class activation map (Grad-CAM) result

**Figure 2:**
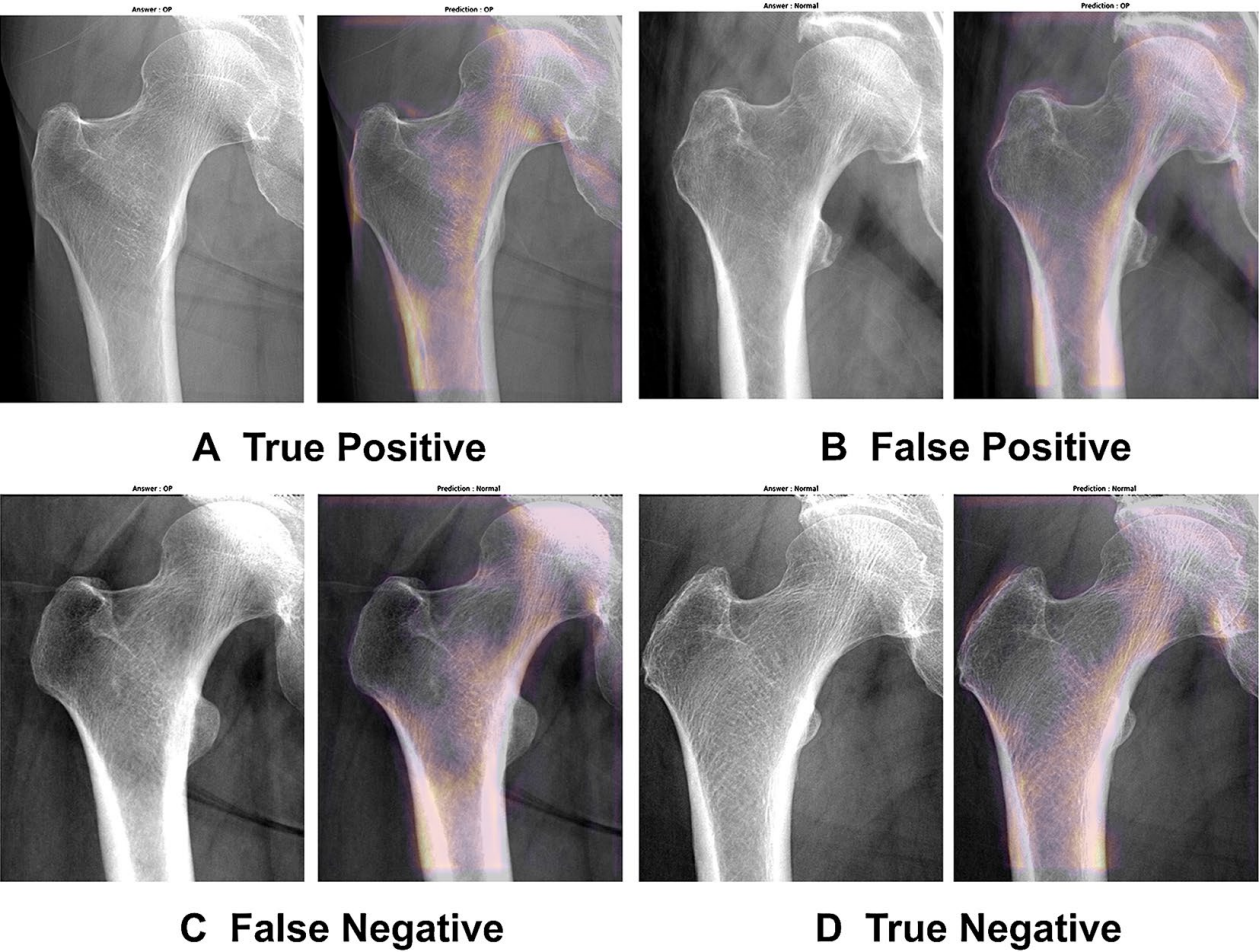
Visualization of the region of interest that our final neural network model interpreted based on Grad-CAM results. The comparison of cropped X-ray images (left) and Grad-CAM result that overlapped on original images (right). The case of true positive that predicted osteoporosis in a patient with real osteoporosis is shown in (**A**), false positive in (**B**), false negative in (**C**), and true negative in (**D**).

All Grad-CAM results from the 101 test sets were confirmed as appropriate by two orthopedic surgeons by perfect agreement (κ = 1.000). Moreover, to predict osteoporosis, we illustrated the Grad-CAM result for true positive, false positive, false negative, and true negative (Fig. 2). Not only the true positive and true negative results but also all false positive and false negative Grad-CAM results appropriately matched not only the cortex line but also the trabecular patterns of proximal femur radiographs, which indicates the validity of our classification model (see Supplementary File S1 online).

### External validation

The confusion matrix of the external validation cohorts from 117 datasets is shown in Table 2. The performance of the model showed an overall accuracy of 71.8%, sensitivity (recall) of 83.7%, and specificity of 38.7%. The PPV, which indicates “precision,” was 79.1%, and the NPV was 46.2%. The AUC value was 0.700, which indicates acceptable performance^10^ (Fig. 1). We also confirmed Grad-CAM results from all 117 datasets to visually verify the model performance (see Supplementary File S2 online).

**Table 2:** Confusion matrix of the external validation.

	Predicted OP	Predicted nonOP	Total
Real OP	72	14	86
Real nonOP	19	12	31
Total	91	26	117

*OP* osteoporosis.

## Discussion

**Figure 3:**
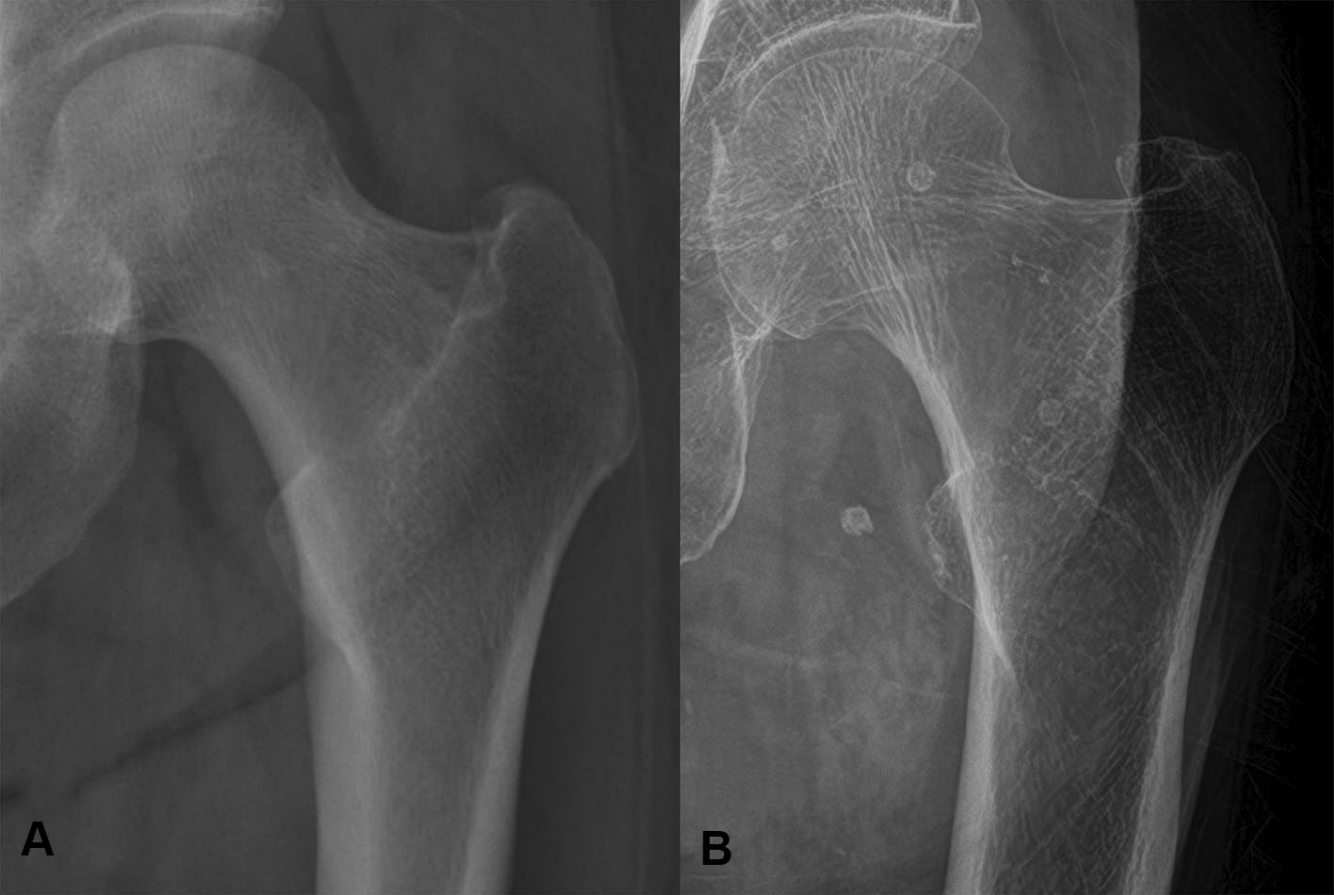
The comparison between normal hip radiographs and osteoporosis. Normal hip radiograph of a 57-year woman, with confirmed T-score of − 0.7, is shown in (**A**), and the hip radiograph of 84-year woman who was diagnosed with osteoporosis with T-score of − 4.6 is shown in (**B**).

DXA, which is regarded as the gold standard of osteoporosis diagnosis, uses the spectral imaging with measurement of the differences of energy levels from two X-ray beams^11^. On the contrary, from the single hip radiographs, decreased BMD can be appreciated by decreased cortical thickness and loss of bony trabeculae in the early stages in simple radiographs (Fig. 3). Indeed, several previous studies in both orthopedic and other various clinical fields have reported that the cortical thickness or trabecular pattern could predict the BMD^9,12^. Therefore, herein, we hypothesized that if we used a deep learning methodology, we could predict the presence of osteoporosis using simple hip radiography. Even though the radiation dose of simple hip radiography is higher compared to DXA, in case DXA is not available, especially in developing countries, or patients have already undergone simple hip radiography for other symptoms, the use of simple hip radiography for screening osteoporosis could be a good alternative to DXA.

In this study, we developed a DNN model that could predict osteoporosis only from a single hip radiograph, with > 80% accuracy and > 90% sensitivity with near 70% specificity based on VGG16 equipped with nonlocal neural network (NLNN) model. We believe that the current proposed model with high accuracy and sensitivity could be considered a useful screening tool for the easy diagnosis of osteoporosis in the real-world clinical setting.

Previously, there were several attempts to predict osteoporosis using simple radiography based on machine learning. In 2012, Harrar et al.^13^ compared the multilayer perceptron neural network to other artificial neural networks to predict osteoporosis defined by DXA in their study using calcaneus radiography. Moreover, in the following year, Kavitha et al.^14^ reported the combination method of histogram-based automatic clustering and support vector machine technique for the prediction of osteoporosis diagnosed by DXA using mandibular cortical bone in the fields of dentistry. However, in the clinical setting, the BMD from DXA is usually based on the examination of the lumbar spine and hip region, so there was a fundamental query in performing the validation between DXA on the lumbar spine/hip regions and other bones in the human body. Recently, some authors reported that their studies tried to predict osteoporosis from lumbar spine or hip radiographs. In 2020, Zhang et al.^15^ used a dataset of 1616 lumbar spine radiographs from 808 postmenopausal women and introduced a deep convolutional neural network (CNN) model to classify the osteoporosis, osteopenia, and normal groups. Moreover, Yamamoto et al.^4^ used the deep learning model to classify osteoporosis using 1131 simple hip radiographs, which is similar to our study. However, both studies have some limitations: The former even performed three-class osteoporosis classification with great performance of the training dataset; in the test dataset, they only showed sensitivity of 57.9–89.3% on screening osteoporosis and sensitivity of 50.0–85.3% on screening osteopenia, which showed lack of consistency. Moreover, they have several potential biases from the characteristics of spine radiography, which is more sensitive to degenerative changes compared to the hip region or several overlying structures near the spine axis. There was also a critical query in the latter study by Yamamoto et al.; even though they reported high prediction performance, such as accuracy, precision, recall, and specificity numerically, in visualization of their classification model from Grad-CAM, their heatmap result was not distributed appropriately to the proximal femoral cortex or trabecular structure, around the lesser trochanter without the proximal femur or pelvic bone as they mentioned. They indicated definitely different regions of interest of DXA, even though they diagnosed and labeled osteoporosis/nonosteoporosis in the DXA result. However, compared to these previous studies, we achieved a favorable performance from our DNNs, which could predict osteoporosis from simple hip radiographs, and the visual explanation of our result from Grad-CAM was also appropriately matched with the proximal femur structure. To the best of our knowledge, this is the first study that showed acceptable osteoporosis diagnostic efficacy using a deep learning model suggesting appropriate Grad-CAM results.

In this study, considering the neural network algorithm, CNN is an efficient algorithm for image processing. However, there are some drawbacks when using CNN architecture. The most significant part related to this study is that CNN only focuses on local features, not global features. As CNN uses a filter map for its linear transformation, CNN only sees locally. In this study, the X-ray dose is not modulated during radiography, and there might be a difference between images of soft tissue intensity divided by bone intensity. Therefore, we contemplated on how to overcome the different windowing levels automatically, and NLNN provided part of the solution. As NLNN works by generating one correlation matrix between every pixel, NLNN provides global information to the CNN network. This methodology is known as attention mechanism and used in various algorithms, such as transformer^16^. Without NLNN, we were unable to train the model at all. However, with NLNN, our model showed reasonable performance with high sensitivity.

This study has several limitations. First, when diagnosing osteoporosis in the clinical setting, we usually consider the BMD from DXA on both the lumbar spine and hip region, but we only considered the hip region in this study because of the complexity of the calculation criteria and various conditions of each patient of spine BMD (e.g., for interpretation of spine DXA, there were numerous exceptions that we do not routinely calculate L1 to L4 BMD). The data preprocessing from both the lumbar spine and hip region could make establishing the neural network model more difficult. However, considering the screening tool, the current model could be helpful even though it did not contain data of the lumbar spine. Second, we did not classify the dataset into three classes—“osteoporosis,” “normal,” and “osteopenia”—but only classified them into two classes, “osteoporosis” or “nonosteoporosis.” Classification can be thought of as an algorithm that makes the decision boundary between classes in data manifold. Therefore, the more subtle the decision boundary is, the more difficult the training process becomes. Especially, osteopenia can be considered the gray zone for the classification task, leading to more difficult differentiation of osteoporosis and osteopenia. Moreover, the limitation of the relatively small number of included dataset was another main reason for the difficulty in dividing the data into three classes, including the “osteopenia” group and continuous outcome predicting model, which correlates actual BMD units. However, the natural progression from normal to osteopenia to osteoporosis is a continuous process, and in the clinical setting, we adopted the T-score for diagnosis of osteoporosis, not a BMD unit, and the two-class identification model could be more intuitive in some ways in a real-world clinical setting than three-class identification, as the screening tool of the current neural network model. Third, we only included postmenopausal women in the study, even though, clinically, they have a high risk of osteoporotic fracture. Indeed, recently, there were concerns about both underdiagnosed and undertreated osteoporosis in the older male population because men are typically not part of routinely recommended screening with DXA, so, in the future study, the larger neural network model should contain the data of the male population. Forth, the fundamental question exists for the definition of osteoporosis, on how well DXA reflects the real osteoporosis. Even though the WHO guideline defined osteoporosis as a T-score ≤  − 2.5 in postmenopausal women, DXA is also one of the examination modalities for evaluating BMD, not an absolute index for defining osteoporosis. Therefore, our current neural network model worked well and showed favorable performance to predict osteoporosis; however, these predictions sometimes might be not an actual osteoporosis prediction, and there could be a query that this only means consistency with the result of the DXA. In the future, further studies are needed, showing more accurate performance of both hip and spine radiography, like central DXA, with larger study datasets. Furthermore, comparisons with other examination modalities for osteoporosis, not only BMD-DXA, are required. Lastly, potential concerns regarding model performance might exist due to the discordance of AUC values between the internal and external validation sets, even though both AUC values indicated acceptable model performance^10^. However, we believe this may be due to the domain generalization issue. Moreover, to the best of our knowledge, no previous study has completely resolved the domain generalization issue. A few studies have attempted to solve it from a mathematical perspective, but they also finally handled this issue on a domain-by-domain basis, that is, as a multicenter study^17^. In the current study, we demonstrated the possibility of predicting osteoporosis using simple hip radiographs. Therefore, we assert that, using our proposed method, anyone can implement and train an osteoporosis prediction model using their own dataset.

In conclusion, even though there were some limitations in the study, the current deep learning network model could be a useful screening tool for the easy diagnosis of osteoporosis in the real-world clinical setting with high accuracy and sensitivity.

## Methods

### Study design and patient selection

This study aimed to establish a deep learning algorithm that classifies osteoporosis or nonosteoporosis defined by the T-score of DXA from simple hip radiographs. This study was approved by the Ethics Committee of Institutional Review Board of Asan Medical Center, Seoul, Republic of Korea (IRB No. 2019-1489). The Asan Medical Center ethics committee waived the need for the informed consent due to the retrospective nature of the study, and the analysis used anonymous clinical data. Data cannot be shared publicly because it contains potentially identifying information of each patient. Data are available from the Asan Medical Center Institutional Data Access/Ethics Committee (contact Asan Medical Center Institutional Review Board, Convergence Innovation Bldg. 88, Olympic-ro 43-gil, Songpa-gu, Seoul, Republic of Korea. Website link, http://eirb.amc.seoul.kr/; E-mail, irb@amc.seoul.kr; Phone, + 82-2-3010-7165). Data collection was performed in accordance with the relevant guidelines and regulations of the committee.

We retrospectively reviewed data with both simple hip anterior–posterior (AP) radiographs and DXA examination results of consecutive patients who visited our hip and pelvic trauma and disease clinic based on our outpatient clinic and inpatient pool between January 2010 and February 2019. We only included the data of patients who (1) were aged ≥ 55 years, (2) were female, and (3) underwent both hip AP radiography and DXA within a month. We excluded the data of patients who (1) had hip osteoarthritis, which potentially leads to BMD overestimation; (2) had other diseases with a specific condition that could result in a bias in DXA results, such as osteonecrosis of femoral head, tumorous condition, and calcific tendinitis of the hip; (3) underwent DXA of the operated hip; (4) underwent hip radiography, which contained foreign body materials on the same side of the DXA; and (5) had either radiography or DXA records from other hospitals. All included data, such as simple hip radiographs and records of DXA, were provided by a single center (Asan Medical Center, Seoul, Republic of Korea). Digital X-ray machines (Model: GM85, Samsung Electronics, Seoul, Korea; Model: GR40CW, Samsung Electronics, Seoul, Korea; Model: Discovery XR656, GE Healthcare, WI, USA; Model: Thunder Platform, GE Healthcare, WI, USA; Model: CXDI, Canon Inc., Tokyo, Japan) at 70 to 78 kV and 207 to 500 tube current were used by an experienced X-ray imaging technologist to acquire standard hip radiographs in the study population. BMD measurement by DXA was performed using Lunar Prodigy Advance system (GE Healthcare, WI, USA). We did not exclude the data of patients who had vascular calcification or Foley catheter on X-ray and did not consider hip dysplasia, even if there was no evidence of osteoarthritis progression. Moreover, we did not consider the position of the hip joint on radiography as an exclusion criterion affecting the quality assessment of hip simple radiographs, but this was regarded as normal variation of real-world data. Finally, the data of 1012 patients were collected.

### Dataset preparation

A total of 1012 simple hip radiographs were divided into two groups: the osteoporosis group, defined by a T-score ≤  − 2.5, and the nonosteoporosis group, defined by a T-score >  − 2.5 in DXA. The definition of osteoporosis followed the WHO diagnostic criteria^18^.

Of 1012 subjects, 513 subjects were diagnosed with osteoporosis, and the other 499 subjects were classified as nonosteoporotic. Of 1012 subjects, one had two radiographs (one subject). Except for this one radiograph, one licensed medical doctor (JR) manually cropped the simple hip bone X-ray images for 1011 subjects.

**Figure 4:**
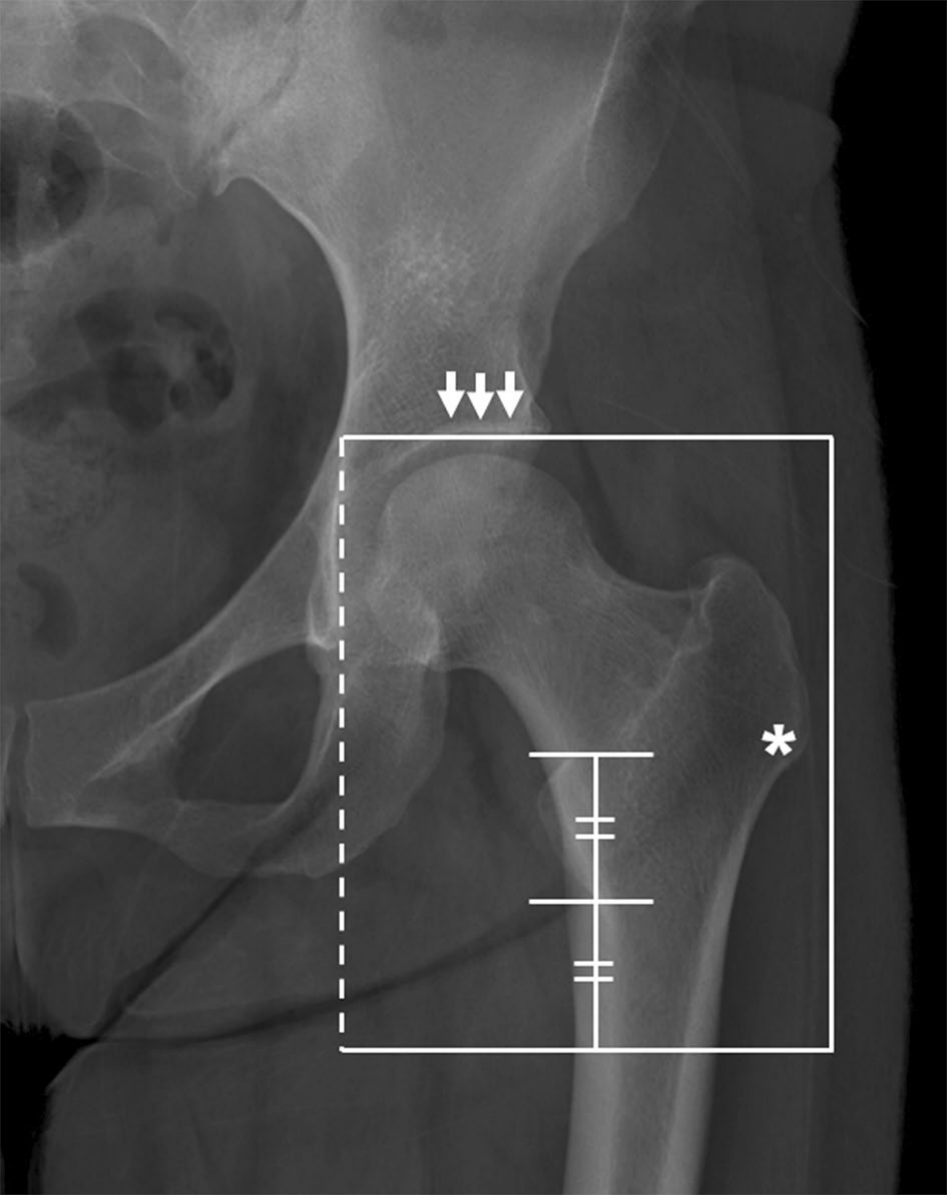
The region of interest that was cropped on simple hip radiograph. The upper border of the cropped image was the acetabular roof (arrows) and femoral head, and the lower border was located as far as the length of the lesser trochanter from the tip of it. The medial border (dotted line) was the crossing of the lateral margin of teardrop, and the lateral border of cropped image was positioned just lateral of the vastus ridge (asterisk).

All images were cropped on the same side where the matched DXA examination was performed. The images were cropped on radiography, which fully contained the proximal femur region similar to the region of interest of DXA, defined as the acetabular roof as the superior border of the cropped region, as the lower margin of the lesser trochanter as the inferior border, lateral margin of the teardrop as the medial border, and lateral of the vastus ridge as the lateral border of the cropped image (Fig. 4).

We were unable to crop 10 images for inappropriate quality of the radiograph. Finally, we have 1001 images that were used for training, validation, and test sets. We randomly selected 80% for training set from entire datasets, and the remaining 10% for validation set and 10% for test set. We used 800 images for the training set (393 osteoporosis; 407 nonosteoporosis), 100 images for the validation set (55 osteoporosis; 45 nonosteoporosis), and 101 images for the test set (56 osteoporosis; 45 nonosteoporosis).

### Training details

For training, the VGG16 network was chosen^19^ as a deep learning model. It is a CNN model that shows high performance. To increase the performance of the network, we not only used the VGG16 network itself but also implemented NLNN^20^ inside the VGG16 network.

When feeding images into the network, due to the nonstandardized image acquiring process of X-ray, we normalized X-ray images with Z-transform with 2-sigma, which translates mean to 0, and then divided with 2-sigma. Furthermore, we randomly mentioned 2-sigma again with 1-sigma to make the model meet various windowing levels. In the mathematical term, we can express our Z-transform as

$${z}_{ij}=\frac{{x}_{ij}-\mu }{2\sigma \pm \epsilon \cdot \sigma }$$

where $\epsilon$ is drawn from a random uniform distribution. After Z-transformation, we indicated pixel values that are bigger than 1 as 1 and smaller than − 1 as − 1.

Moreover, we used the data augmentation technique. For data augmentation, five strategies were used: original imaging, blurring, sharpening, shearing, and rotation with a small angle. As there were five strategies, we set the number of iterations per epoch to be 4,000 iterations, which are 800 images multiplied by five strategies.

Additionally, we used Keras framework of Python language, with TensorFlow backend. Loss was set to be vanilla binary cross entropy, and we trained the model for 300 epochs. We selected the optimizer to be Adam^21^ with a learning rate of ${10}^{-6}$. After every epoch, validation loss and validation accuracy were calculated, and the model with the best validation accuracy was selected for the final model.

### Model evaluation

After training, we calculated the confusion matrix to validate model performance, and from this, we evaluated the accuracy, sensitivity, specificity, PPV, and NPV. Moreover, we also drew the ROC curve and calculated the AUC. Furthermore, we used a Grad-CAM^22^ overlapping the original image to visualize the model performance. The Grad-CAM is a tool of visual explanations of DNNs, activating the mapping that the deep learning network is seeing. Thus, Grad-CAM is a tool for explainable AI. Through Grad-CAM, one can visualize where a deep learning algorithm sees using a gradient of deep learning algorithm at the image level. All Grad-CAM results of the 101 test sets were independently reviewed by two board-certified orthopedic surgeons who are the faculty of orthopedic hip and pelvis (KCH and YPW). The adequacy of Grad-CAM were evaluated by the distribution of the heatmap, through not only the cortex line but also the trabecular patterns of the proximal femur, which is checked as binary data (yes/no) for adequacy. The agreement between reviewers was correlated with kappa values a priori: κ = 1, corresponding to *perfect* agreement; 1.0 > κ ≥ 0.8, *almost perfect* agreement; 0.8 > κ ≥ 0.6, *substantial* agreement; 0.6 > κ ≥ 0.4, *moderate* agreement; 0.4 > κ ≥ 0.2, *fair* agreement; and κ < 0.2, *slight* agreement.

### External validation

The neural network model was externally validated with the data that were prospectively collected from another university hospital between October 2020 and July 2021. Digital X-ray machines (Model: Innovision DXII, DK Medical, Seoul, Korea; Model: Discovery XR656, GE Healthcare, WI, USA) at 70 to 78 kV and 200 to 500 tube current were used in hip radiography, and BMD measurement by DXA was performed using Lunar Prodigy Advance system (GE Healthcare, WI, USA). A total 117 datasets (86 osteoporosis; 31 nonosteoporosis) were used for external validation. The overall accuracy, sensitivity, specificity, PPV, and NPV were calculated. The ROC curve and AUC value were calculated. We also used Grad-CAM to visually verify the model performance.

### Ethical approval and informed consent

This study was approved by the Institutional Review Board of Asan Medical Center, Seoul, Republic of Korea (IRB No. 2019-1489), informed consent was waived due to the retrospective nature of the study, and the analysis used anonymous clinical data.

### Consent for publication

All authors agreed to publish this manuscript.

## Supplementary Information

Supplementary Figure 1.

Supplementary Figure 2.

## Data Availability

The data presented in this study are available on request from the corresponding author. The data are not publicly available due to conditions of the ethics committee of our university.
