# Supplementary figures and images for: Prediction of osteoporosis from simple hip radiography using deep learning algorithm
Source: Sci Rep. 2021 Oct 7;11:19997. doi: 10.1038/s41598-021-99549-6 (PMC8497544; doi:10.1038/s41598-021-99549-6)

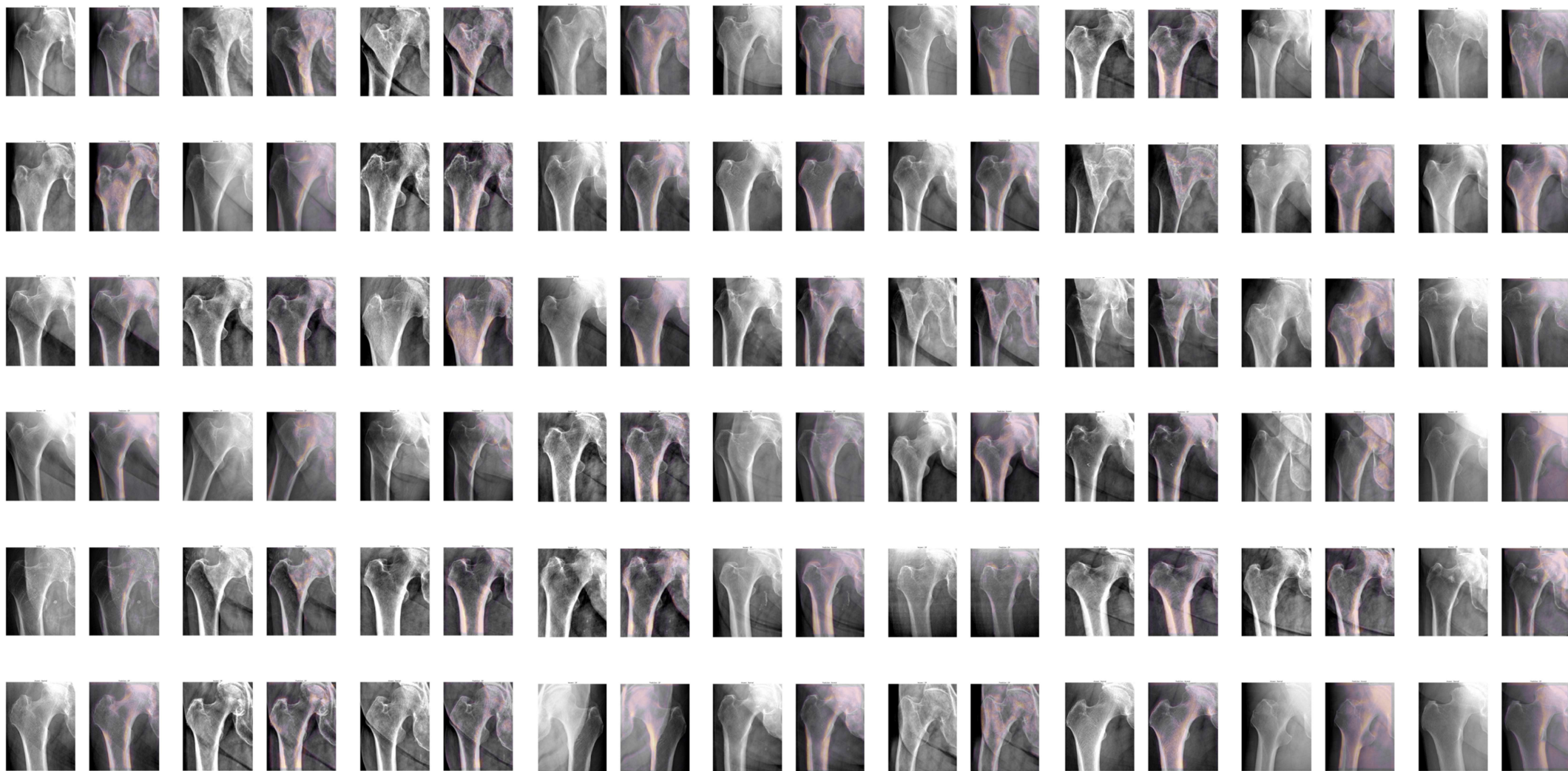

Supplementary File S1. Grad-CAM results of all 101 test sets from original data

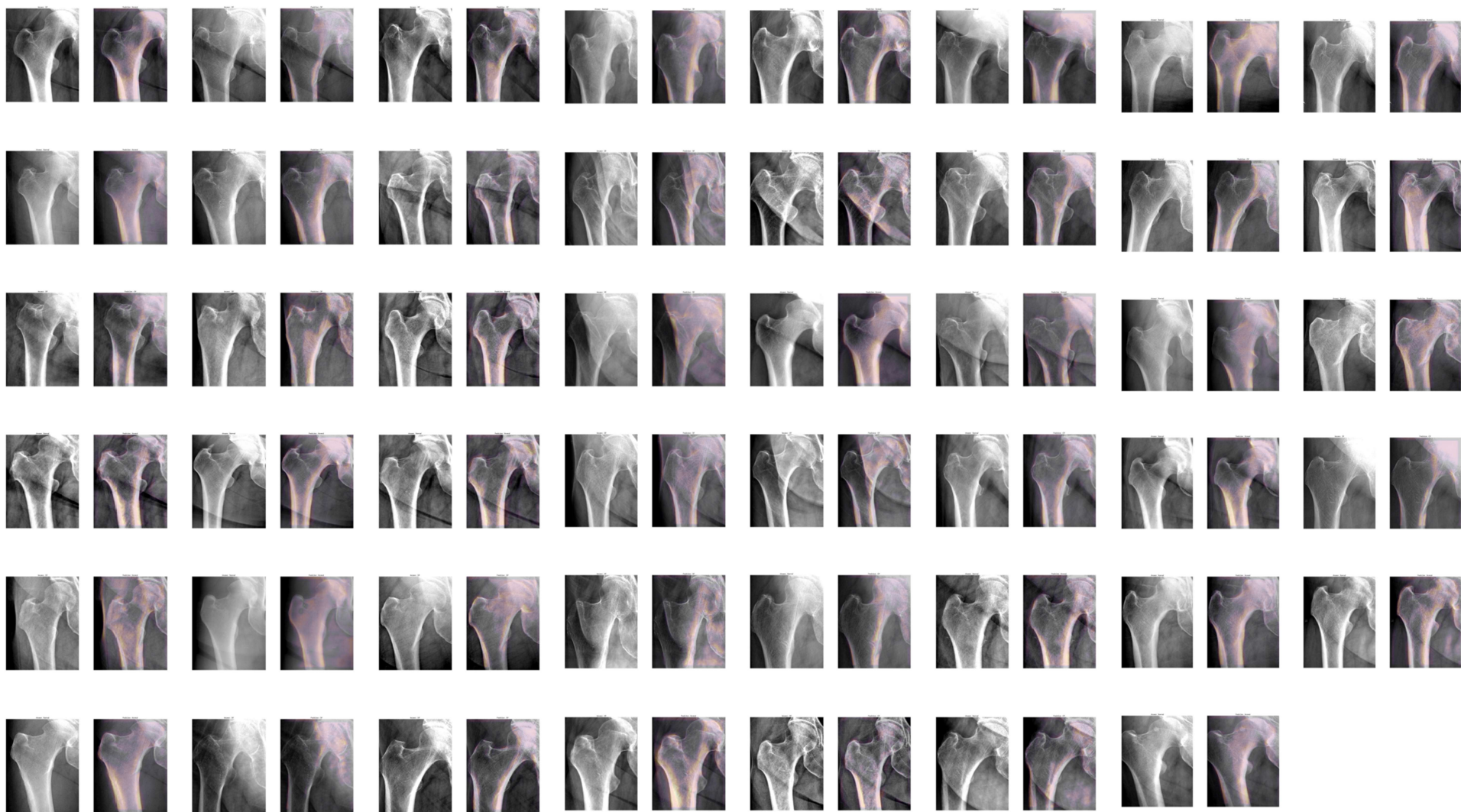

Supplement: Supplementary file 1 — Supplementary Figure 1. [file 41598_2021_99549_MOESM1_ESM.pdf]

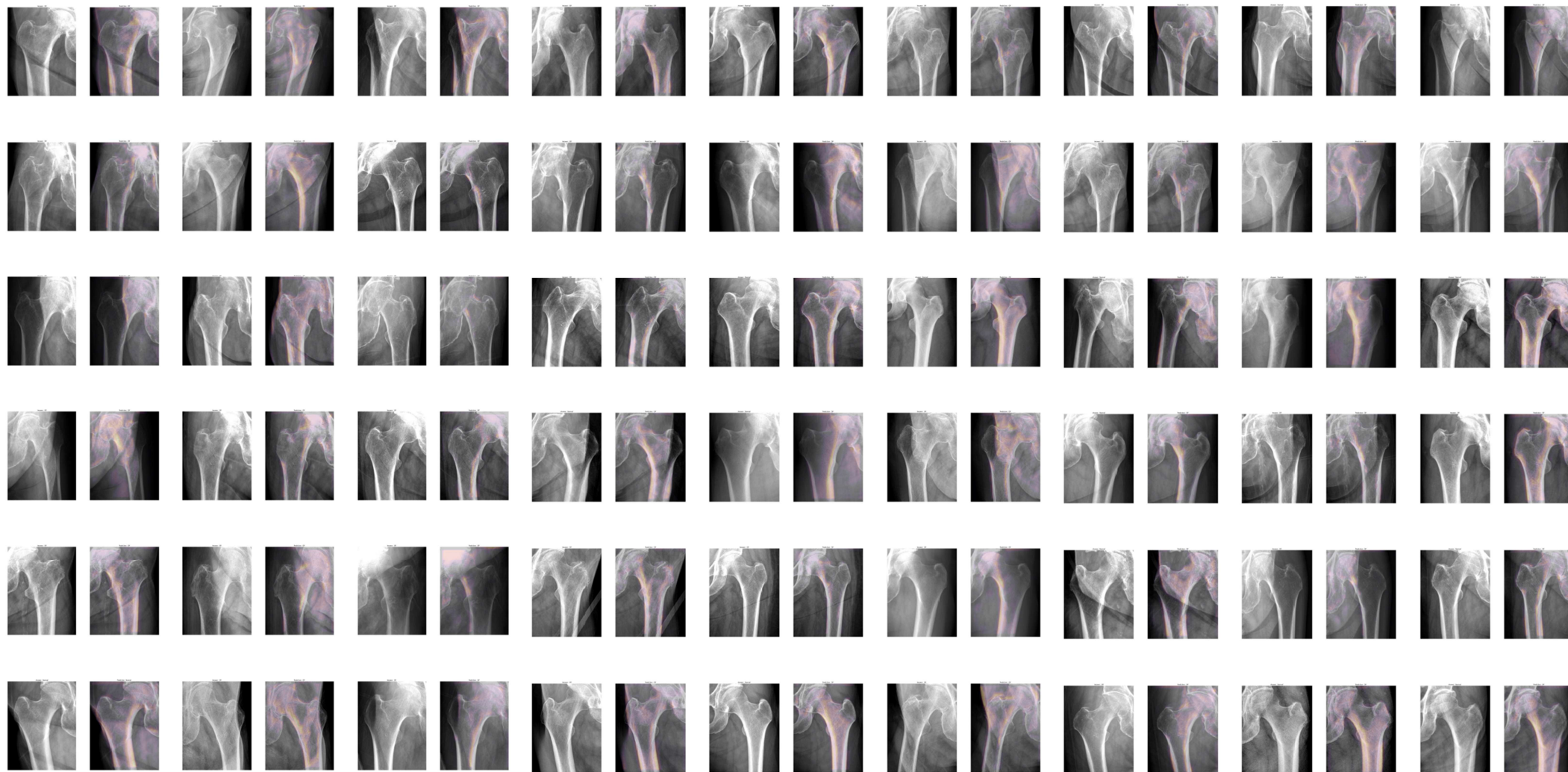

Supplementary File S2. Grad-CAM results of all 117 external validation sets

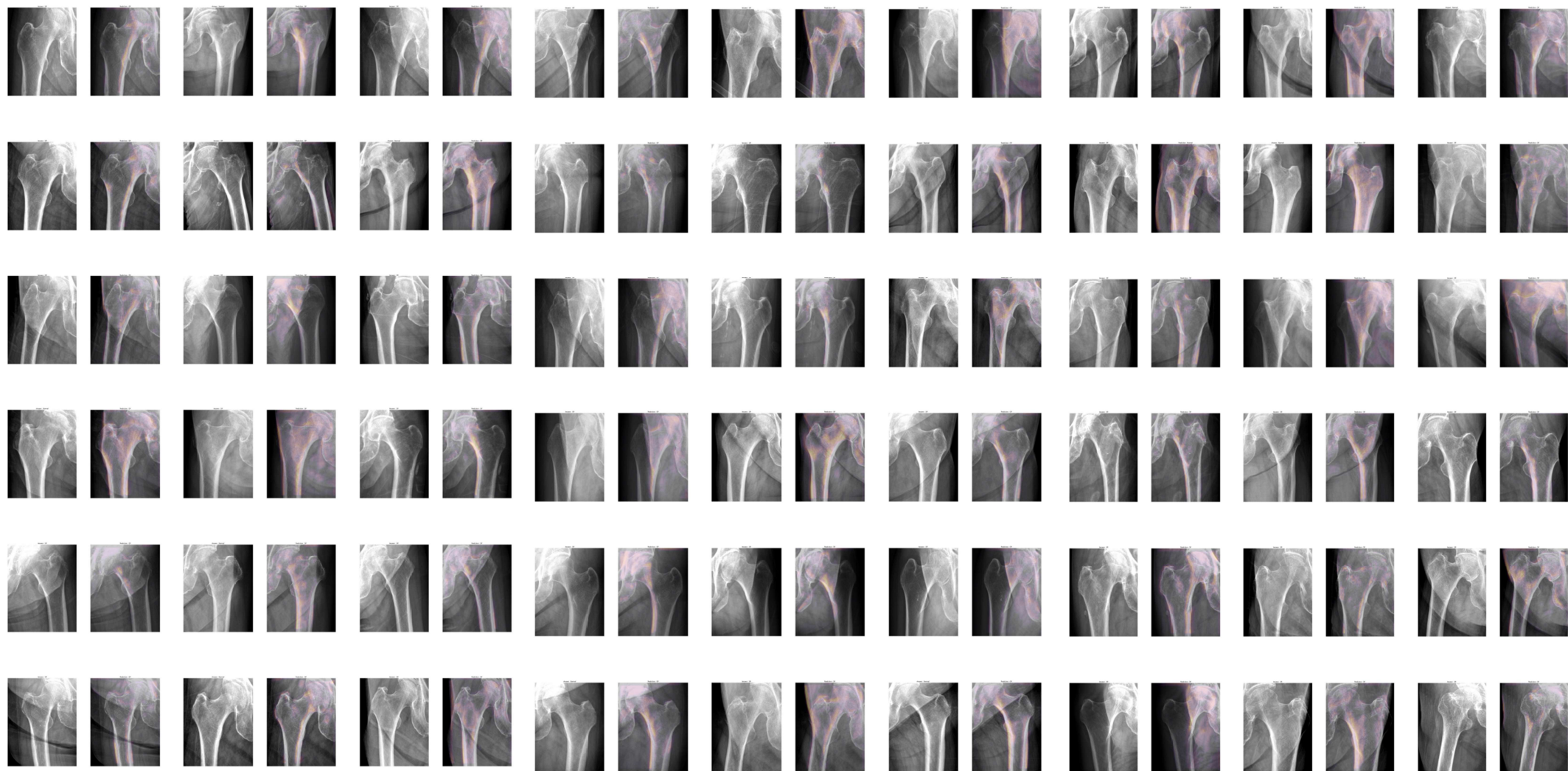

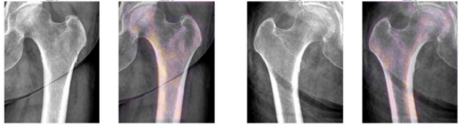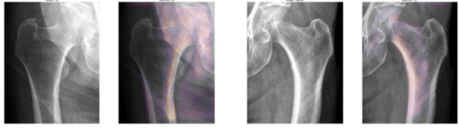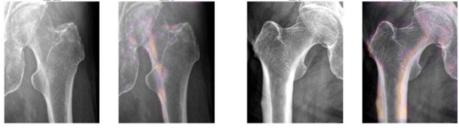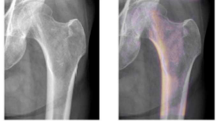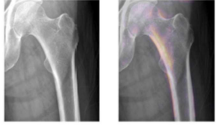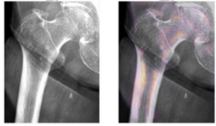

Supplement: Supplementary file 2 — Supplementary Figure 2. [file 41598_2021_99549_MOESM2_ESM.pdf]
